# Lack of Hyperinhibition of Oriens Lacunosum-Moleculare Cells by Vasoactive Intestinal Peptide-Expressing Cells in a Model of Temporal Lobe Epilepsy

**DOI:** 10.1523/ENEURO.0299-21.2021

**Published:** 2021-12-23

**Authors:** Megan Wyeth, Paul S. Buckmaster

**Affiliations:** Department of Comparative Medicine, Stanford University, Palo Alto, CA 94305-5410

**Keywords:** CA1, CCK, hippocampus, interneuron, OLM, VIP

## Abstract

Temporal lobe epilepsy remains a common disorder with no cure and inadequate treatments, potentially because of an incomplete understanding of how seizures start. CA1 pyramidal cells and many inhibitory interneurons increase their firing rate in the seconds-minutes before a spontaneous seizure in epileptic rats. However, some interneurons fail to do so, including those identified as putative interneurons with somata in oriens and axons targeting lacunosum-moleculare (OLM cells). Somatostatin-containing cells, including OLM cells, are the primary target of inhibitory vasoactive intestinal polypeptide and calretinin-expressing (VIP/CR) bipolar interneuron-selective interneurons, type 3 (ISI-3). The objective of this study was to test the hypothesis that in epilepsy inhibition of OLM cells by ISI-3 is abnormally increased, potentially explaining the failure of OLM recruitment when needed most during the ramp up of activity preceding a seizure. Stereological quantification of VIP/CR cells in a model of temporal lobe epilepsy demonstrated that they survive in epileptic mice, despite a reduction in their somatostatin-expressing (Som) cell targets. Paired recordings of unitary IPSCs (uIPSCs) from ISI-3 to OLM cells did not show increased connection probability or increased connection strength, and failure rate was unchanged. When miniature postsynaptic currents in ISI-3 were compared, only mIPSC frequency was increased in epileptic hippocampi. Nevertheless, spontaneous and miniature postsynaptic potentials were unchanged in OLM cells of epileptic mice. These results are not consistent with the hypothesis of hyperinhibition from VIP/CR bipolar cells impeding recruitment of OLM cells in advance of a seizure.

## Significance Statement

Inadequate recruitment of inhibitory cells in general, and oriens and axons targeting lacunosum-moleculare (OLM) cells in particular, may be a mechanism of seizure initiation, making it important to determine why OLM cells do not fire faster and provide preictal feedback inhibition when presynaptic CA1 pyramidal activity is ramping up. This study excludes aberrantly increased inhibition of OLM cells by vasoactive intestinal polypeptide (VIP) bipolar cells as the cause, pointing to other possibilities for investigation.

## Introduction

A critical question in epilepsy research is how seizures emerge from an epileptic network. Recordings from a rat model of temporal lobe epilepsy indicate that in the seconds-minutes before a spontaneous seizure granule cells in the dentate gyrus and pyramidal cells in CA1 and subiculum increase their firing rate (Fujita et al., 2014; but see Ewell et al., 2015). Correspondingly, the majority of interneurons in the dentate gyrus, CA1 and the subiculum also increase their firing rate in advance of a seizure (Toyoda et al., 2015). However, interneurons putatively identified as somatostatin-expressing (Som) cells with somata in oriens and axonal projections to lacunosum-moleculare (OLM), failed to increase their firing rate (Toyoda et al., 2015), this at a time when responsive inhibition may be critical to reign in activity. This apparent failure of OLM cells potentially contributes to seizure-initiation, and raises the question of what is responsible for the deficient engagement of OLM cells despite increased firing in CA1 pyramidal cells, their primary source of excitatory drive (Lacaille et al., 1987; Ali and Thomson, 1998; Pouille and Scanziani, 2004).

The GABAergic system undergoes remarkable plasticity to complex effect following an initial insult in acquired temporal lobe epilepsy (Scharfman and Brooks-Kayal, 2014). Of all neuronal types, some of the best evidence is for a myriad of changes to Som interneurons. Som cells are among the vulnerable neuron populations that incur cell death in patients and animal models (Sloviter, 1987; de Lanerolle et al., 1989; Robbins et al., 1991; Dinocourt et al., 2003). However, some Som cells survive, especially in CA1 (de Lanerolle et al., 1989), and undergo extensive axon sprouting (Zhang et al., 2009), innervating abnormal targets in the case of CA1 OLM cells (Peng et al., 2013). The striking changes to Som cells make them of particular interest to understanding the mechanisms underlying epilepsy. The transcription factor Distal-less homeobox 1 (*Dlx1*) is required for the longevity of a subset of interneurons in the adult neocortex and hippocampus; *Dlx1* knock-out mice show a selective loss of Som and calretinin-expressing (CR) cells and develop mossy fiber sprouting and recurrent seizures, supporting the relevance of Som cells to epilepsy (Cobos et al., 2005). Furthermore, activation of cholinergic projections from the medial septum recruited CA1 Som interneurons to delay kindling and reduce seizure severity (Wang et al., 2020). Optogenetically silencing distal-dendrite targeting Som cells enhanced the mean firing rates of pyramidal cells to a similar degree as silencing parvalbumin-expressing interneurons, however silencing Som cells also increased burst firing in CA1 pyramidal cells (Royer et al., 2012). Increased pyramidal cell bursting because of depolarized dendrites may facilitate seizure initiation (Traub and Llinás, 1979; Jensen and Yaari, 1997; Truccolo et al., 2011).

**Figure 1.**
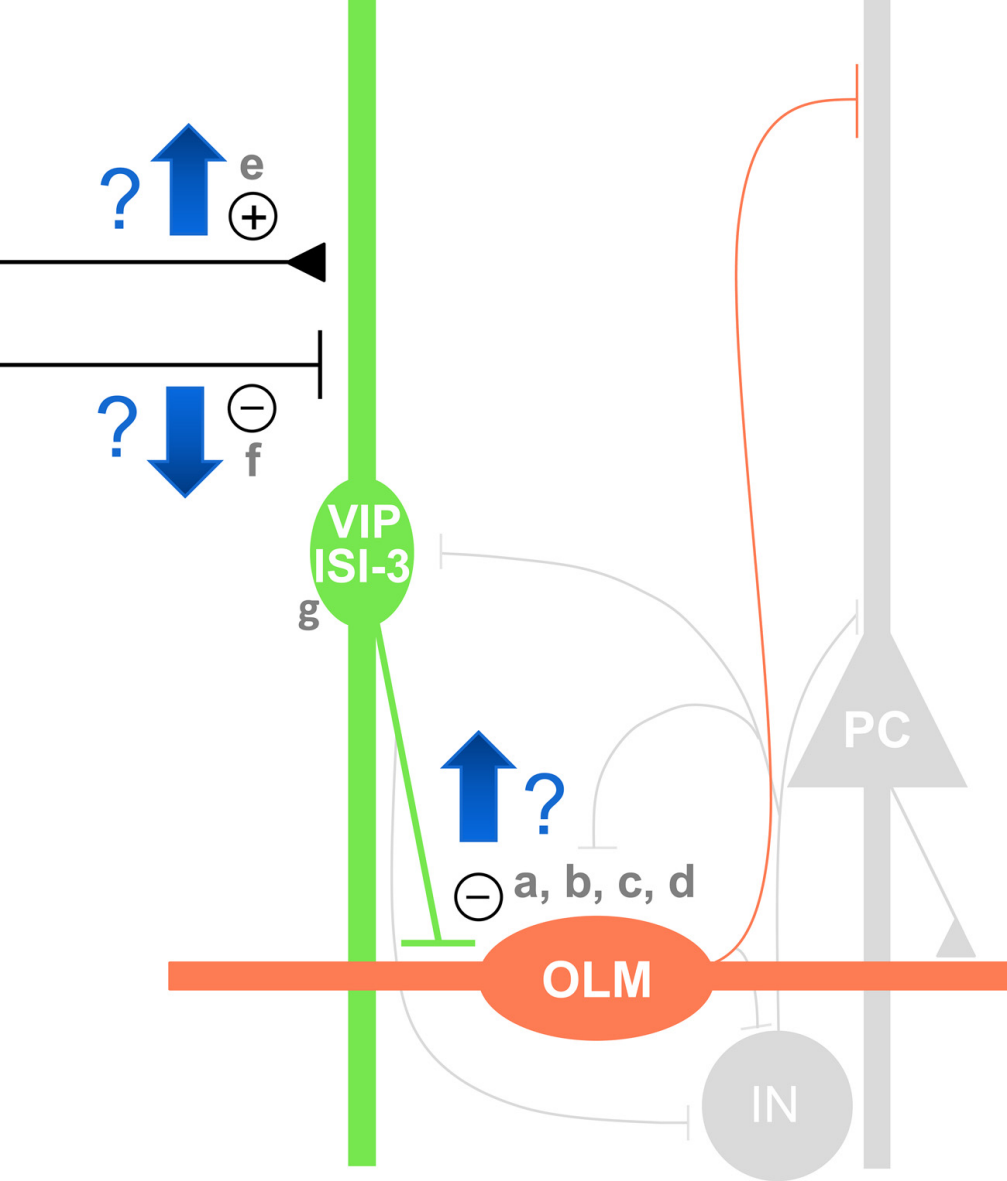
Possible mechanisms for hypothesized increased inhibition of somatostatin-containing OLM cells by VIP ISI-3 in CA1. a, Increased probability of ISI-3 synapsing with OLM cells because of OLM cell loss and/or VIP axonal sprouting. b, Increased amplitude of uIPSCs between ISI-3 and OLM cells. c, Reduced failure rate at ISI-3 to OLM cell synapses. d, Increased synaptic summation at ISI-3 to OLM cell connections. e, Increased excitatory synaptic drive of ISI-3. f, Reduced synaptic inhibition of ISI-3. g, Increased excitability of ISI-3 (PC: glutamatergic pyramidal cell, IN: other GABAergic interneurons in the local circuit).

This study was undertaken to address why an epileptic circuit may fail to recruit OLM interneurons. Recent work demonstrated no reduction in the characteristic facilitation of excitatory inputs to OLM cells in the rat pilocarpine model of temporal lobe epilepsy (Pothmann et al., 2019). Here, we test the complementary possibility that inhibition of OLM cells may be aberrantly increased in temporal lobe epilepsy, undercutting excitatory drive from CA1 pyramidal cells (Fig. 1). Type 3 interneuron-selective interneurons (ISI-3), which express vasoactive intestinal peptide and CR (VIP/CR), are a major source of inhibition to OLM cells (Acsády et al., 1996a,b; Chamberland et al., 2010; Tyan et al., 2014) and regulate the timing and rate of OLM cell firing (Tyan et al., 2014). With dendrites spanning the depth of CA1 (Acsády et al., 1996a,b), VIP bipolar cells are positioned to receive inputs from the same pathways that drive CA1 pyramidal cells. VIP-expressing interneurons are among the populations that survive in patients with temporal lobe epilepsy (de Lanerolle et al., 1995); however, the VIP/CR population undergoes some of the largest transcriptomic changes among interneurons (Pfisterer et al., 2020). While ISI-3 inhibitory inputs to OLM cells are of small amplitude and low release probability in control mice (Tyan et al., 2014), we hypothesized they may be stronger in mice with chronic seizures. This could allow VIP cells to open a large rift, rather than a narrow gap, in the blanket of inhibition provided by Som cells (Karnani et al., 2016a). Paired recordings allow direct interrogation of the ISI-3 to OLM cell connection.

The objectives of this study were to investigate changes to the recruitment of ISI-3 and their inhibition of OLM interneurons in a mouse model of temporal lobe epilepsy to test the overall hypothesis that excessive inhibition from VIP cells results in deficient synaptic recruitment of OLM cells, thereby contributing to seizure onset.

## Materials and Methods

### Animals and pilocarpine treatment

CD1 mice heterozygous for VIP-eGFP were kindly provided by Lisa Topolnik (Université Laval; RRID:MMRRC_031009-UCD) and housed in a 12/12 h light/dark cycle. The GFP expression in these mice and the properties of hippocampal ISI-3 have been well-characterized (Chamberland et al., 2010; Tyan et al., 2014; Luo et al., 2020). GFP expression in pups was confirmed using a DNeasy kit (QIAGEN, 250). At five to six weeks male and female mice were treated with 1 mg/kg scopolamine or atropine methyl bromide (muscarinic receptor antagonists), intraperitoneally, to block peripheral effects, followed after 30 min by 250–290 mg/kg pilocarpine (muscarinic receptor agonist), intraperitoneally. Mice were continuously observed for status epilepticus and 2 h after the first behavioral seizure were injected with 5 mg/kg diazepam (GABA receptor agonist) to suppress seizures, and with lactated ringer’s to replace lost fluids. Mice were reinjected with diazepam 1 h later if there was still evidence of status epilepticus. Mice that did not experience status epilepticus were used as controls, along with naive mice. After a weeklong recovery, mice that experienced status epilepticus were video-monitored 10 h/d until they developed spontaneous seizures (first observed seizure 6–171 d postpilocarpine, median 30 d, *n* = 99). Mice were group-housed unless they became aggressive, in which case they were housed individually. All animal procedures were performed in accordance with the Stanford University animal care committee’s regulations.

### Immunohistochemistry

Mice were euthanized by pentobarbital overdose and perfused through the ascending aorta with 4% paraformaldehyde (PFA) with a volume (ml) twice their body weight (g). Brains were left *in situ* for 1 h at 4°C, postfixed 1 h in 4% PFA, rinsed thoroughly in 0.1 m phosphate buffer, and equilibrated in 30% sucrose. Hippocampi were isolated and sectioned at 40 μm on a sliding microtome and stored in immuno-cryoprotectant (25% glycerol, 30% ethylene glycol in 0.1 m phosphate buffer) at −20°C until use. In preparation for immunohistochemistry, sections were thoroughly rinsed in 0.1 m PBS, pretreated with 1% H_2_O_2_ for 30 min, rinsed and incubated in blocking solution containing 3% normal goat serum (NGS), 2% bovine serum albumin (BSA) and 0.3% triton in 0.1 m Tris-buffered saline (TBS) for 3 h at room temperature (RT).

For double immunofluorescent labeling against GFP and CR, rinsed sections were incubated in rabbit anti-CR (Swant, 7697) at 1:30,000 and chicken anti-GFP (Aves, GFP-1020) at 1:30,000 in 0.1 m TBS with 1% NGS, 0.2% BSA, and 0.3% triton over seven nights at 4°C. Sections were rinsed and incubated in secondary solution containing 488 anti-chicken and 555 anti-rabbit (Invitrogen, A11039 and A21428) at 1:500 in 0.1 m TBS with 3% NGS and 0.3% triton for 5 h at RT. Labeled sections were mounted and coverslipped with Prolong Gold antifade reagent (Invitrogen, P36930).

For immunohistochemistry against Som, following rinsing, sections were incubated in rabbit anti-somatostatin (Peninsula Labs, T4130) at 1:25,000 with 1% NGS, 0.2% BSA and 0.3% triton in 0.1 m TBS over 7 nights at 4°C. Sections were rinsed and incubated in goat anti-rabbit secondary at 1:500 with 2% BSA in TBS for 2 h RT, followed by rinsing and 2 h in Vectastain Elite ABC solution at 1:500 (Vector Labs, PK-6100). For the chromogenic reaction, sections were incubated for 5 min in 3,3'-diaminobenzidine (DAB) solution containing 2% DAB, 0.04% NH_4_Cl, and 0.015% glucose oxidase in 0.1 m Tris buffer, followed by 10 min in the same solution with the addition of 0.1% β-D-glucose. Stained sections were mounted, dehydrated, and coverslipped for analysis.

### Stereology

One hippocampus from each mouse was analyzed using the optical fractionator method (11 controls and 11 epileptic;(West et al., 1991). Commencing with a random section, every 12th section along the length of the hippocampus was labeled for quantification (10–12 sections per mouse). The investigator performed the analysis without reference to the group designation of the tissue. To quantify the number of somatostatin-labeled interneurons, CA1 oriens and alveus were first outlined using a 10× objective (Neurolucida, MBF Bioscience). Using a 100× objective, all profiles not cut at the superficial surface were counted (345 profiles/hippocampus on average). These counts were extrapolated to estimate the number of cells per hippocampus. For somatostatin-labeled interneuron quantification the mean coefficient of [within mouse] error (0.07) was less than half of the coefficient of variation across all mice (0.17) confirming that less than half of group variance was because of the within animal estimation procedure. Similarly, to quantify the number of VIP and CR-labeled interneurons, every VIP/CR double-labeled cell in the CA1 radiatum and pyramidal cell layers that was not cut at the superficial surface was counted in each section using a 40× objective (124 profiles/hippocampus on average). Again, the counts were extrapolated to estimate the number of cells per hippocampus. For VIP+/CR+ interneurons the mean coefficient of error (0.09) was less than a third of the coefficient of variation (0.29), and for VIP+/CR– interneurons the mean coefficient of error (0.11) was less than a quarter of the coefficient of variance (0.45), indicating sufficient sampling.

### Slice electrophysiology

Mice [postanal day (P)22–P141, median: P86] were deeply anesthetized with isofluorane, decapitated and 300 μm coronal sections taken on a vibratome (Leica VT1000S) in ice-cold oxygenated NMDG solution (135 mm *N*-methyl-D-glucamine, 10 mm D-glucose, 1.5 mm MgCl_2_, 1.2 mm KH_2_PO_4_, 1.0 mm KCl, 0.5 mm CaCl_2_, and 20 mm choline bicarbonate; pH 7.4, 300–305 mOsm). Sections recovered for 20 min at 32°C in oxygenated partial sucrose (85 mm NaCl, 55 mm sucrose, 26 mm NaHCO_3_, 25 mm glucose, 4 mm MgCl_2_, 2.5 mm KCl, 1.25 mm NaH_2_PO_4_-H_2_O, and 0.5 mm CaCl_2_; pH 7.4, 300–305 mOsm), then were transferred to oxygenated artificial CSF (ACSF; 130 mm NaCl, 24 mm NaHCO_3_, 10 mm D-glucose, 3.5 mm KCl, 1.25 mm NaH_2_PO_4_-H_2_O, 2.5 mm CaCl_2_, and 1.5 mm MgCl_2_; pH 7.4, 300–305 mOsm) for storage at RT until use. In the recording chamber sections were perfused at 3 ml/min with ACSF warmed to 32°C by a temperature controller (Warner Instruments, TC-344B). Borosilicate electrodes were pulled to a resistance of 4–6 MΩ on a micropipette puller (Sutter Instruments, P-97). Cells were visualized with a 40× objective and a Hamamatsu camera; interneurons expressing eGFP were identified with blue light. Whole-cell voltage clamp recordings were made with cesium gluconate intracellular solution (95 mm Cs-gluconate, 5 mm CsCl, 0.6 mm EGTA, 4 mm NaCl, 40 mm HEPES, 5 mm MgCl_2_, 2 mm Na_2_ATP, 0.3 mm NaGTP, 1 mm QX-314, and 20 mm biocytin; pH 7.3, 295–300 mOsm) on an Axopatch 200B (Molecular Devices). Signals were digitized at 20 kHz on a Digidata 1400A (Molecular Devices, filtered at 1 kHz) controlled by pClamp 10.6 software (Molecular Devices). The liquid junction potential (−7 mV) was not corrected. Action potential frequency was recorded before membrane rupture for whole-cell recordings. Passive properties were recorded on breaking into the cell and recordings with access resistance above 20 MΩ or that varied >15% were discarded. Miniature potentials were isolated with tetrodotoxin (TTX; sodium channel blocker, 1 μm) and postsynaptic currents were found using a template search (averaged from at least 100 events). Current clamp recordings were made using potassium gluconate intracellular solution (130 mm K-gluconate, 10 mm HEPES, 2 mm MgCl_2_, 2 mm Na_2_ATP, 0.3 mm NaGTP, 0.6 mm EGTA, and 20 mm biocytin; pH 7.4, 290–300 mOsm) on an Axopatch 1D (Molecular Devices). Paired recordings were performed holding presynaptic VIP/CR bipolar cells in current clamp and producing trains (10 pulses at 10–100 Hz) or pairs (20 Hz) of action potentials every 5 s with 1.2 ms, 2-nA current injections while holding the postsynaptic OLM cell in voltage clamp. Unitary IPSC (uIPSC) properties were analyzed by averaging at least 20 consecutive traces, and amplitudes reflect the average of the first peak amplitude including failures unless otherwise noted.

### Immunofluorescence on recorded sections

For *post hoc* labeling, sections were drop-fixed in 4% PFA over 1–4 nights at 4°C. After thorough rinsing sections were incubated in 5% triton with 10% NGS in 0.1 m TBS overnight at RT, followed by three nights in primary solution containing chicken anti-GFP (1:1000, Aves, GFP-1020) and rabbit anti-somatostatin (1:5000, Peninsula Labs, T4103) with 1:50 NGS in 0.1 m TBS. Rinsed sections were incubated in secondary solution containing 488 anti-chicken, 555 streptavidin, and 633 anti-rabbit (1:500, Invitrogen) with 1:30 NGS in 0.1 m TBS. Finally, sections were rinsed in 0.1 m phosphate buffer, mounted and coverslipped with Prolong Gold (Invitrogen, P36930). ISI-3 VIP bipolar cells with somata in CA1 radiatum and pyramidale were identified by their characteristic bidirectional dendrites spanning oriens and ramifying in lacunosum-moleculare along with their distinctive axon targeting oriens (Acsády et al., 1996a; Freund and Buzsáki, 1996). OLM cells in oriens were identified by their horizontal dendrites with distal spindly spines and distinguishing axon targeting lacunosum-moleculare (Schwartzkroin et al., 1990; McBain et al., 1994; Freund and Buzsáki, 1996). Cells were imaged on a Nikon A1 inverted confocal microscope.

### Analysis

SigmaPlot 12 (Systat Software) was used for statistics and *p* < 0.05 was considered significant. Graphs include individual data points. For group averages, bars illustrate the mean and error bars portray SE. Box plots indicate the median, 25th and 75th percentiles in cases where the data called for nonparametric statistics.

## Results

### VIP/CR interneurons and OLM cell quantification

**Figure 2.**
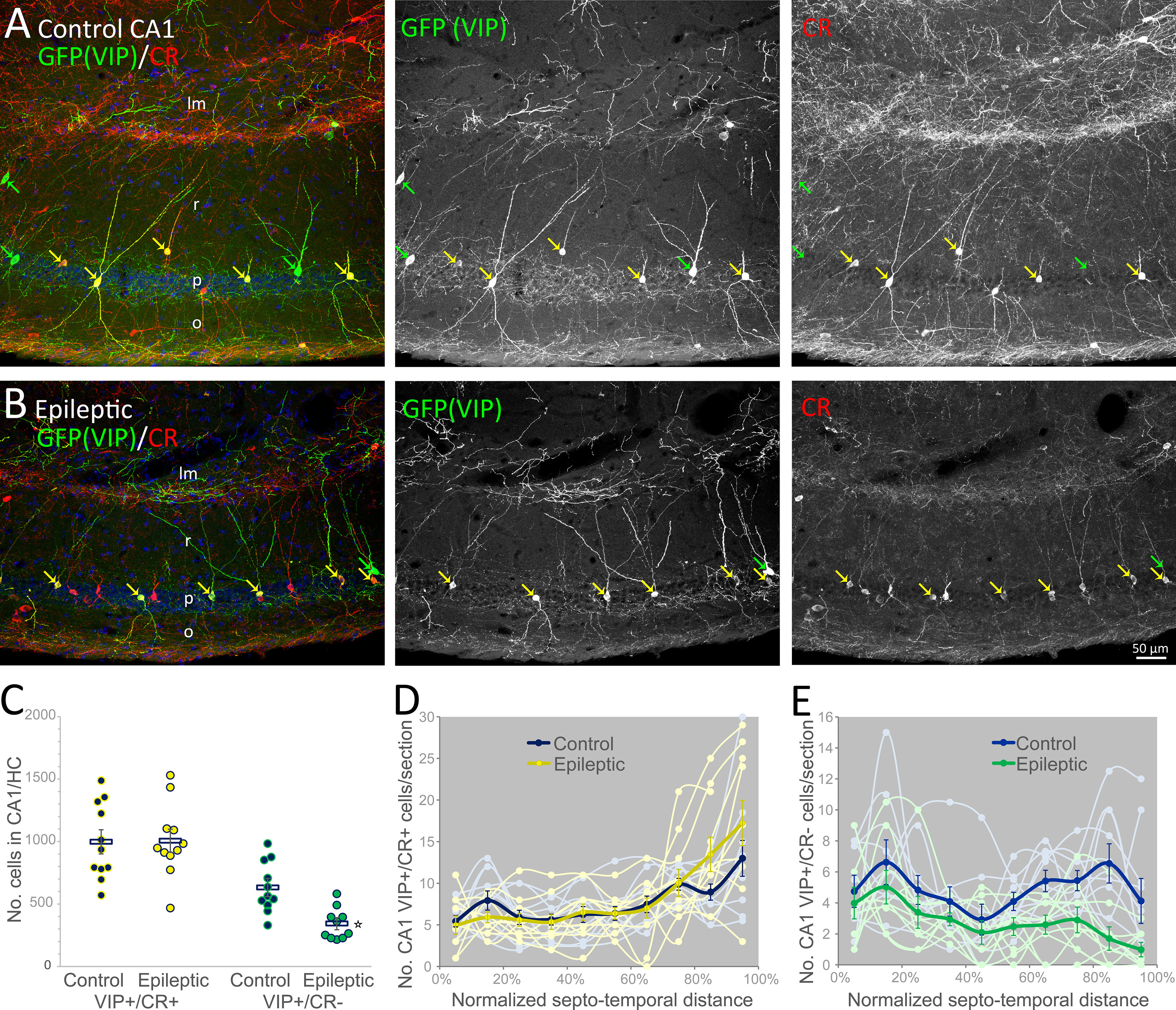
GFP(VIP)+/CR+ cells persist in CA1 of epileptic mice. ***A***, In a control VIP-eGFP mouse, somata are labeled with a GFP antibody (green) in CA1 stratum radiatum (r) and the pyramidal cell layer (p). Interneuron-selective bipolar cells (yellow arrows) additionally express CR (red). GFP(VIP)-expressing cells that lack CR (green arrows) include basket cells that inhibit pyramidal cells. Nuclei are labeled with DAPI (blue). ***B***, CA1 of an epileptic mouse is shrunken, yet GFP(VIP)+/CR+ cells remain evident (yellow arrows). However, there are fewer GFP(VIP)+/CR– cells. ***C***, The number of GFP(VIP)+/CR+ interneurons, and GFP(VIP)+/CR– interneurons in the radiatum and pyramidal cell layers of CA1 in control and epileptic mice were estimated by the optical fractionator method. On average, the number of GFP(VIP)+/CR+ cells per hippocampus (HC) is not reduced in epileptic mice, although the number of GFP(VIP)+/CR– interneurons is approximately halved (*t test: *p* = 0.0006). ***D***, Neither is the number of GFP(VIP)+/CR+ cells reduced compared with controls across the septo-temporal extent of the hippocampus. ***E***, However, the reduction of GFP(VIP)+/CR– interneurons is particularly apparent at temporal levels of the epileptic hippocampus (o: stratum oriens, lm: strata lacunosum-moleculare).

Interneuron classes are variously vulnerable in epilepsy. For ISI-3 to hyperinhibit OLM cells they must survive in chronic epilepsy, potentially at a higher rate than OLM cells. There is evidence that VIP-expressing cells are preserved in reorganized hippocampi from epileptic patients (de Lanerolle et al., 1995) and epileptic mice (David and Topolnik, 2017). Hippocampal VIP-expressing cells fall into several broad categories: ISI-3 bipolar cells in radiatum and pyramidale that co-express CR and target interneurons in oriens (like OLM cells), ISI-2 cells with somata frequently in lacunosum-moleculare that target other interneurons in radiatum, basket cells (that can co-express cholecystokinin), and long-range projection cells in oriens with axonal projections to oriens and subiculum (Acsády et al., 1996a; Francavilla et al., 2018). For stereological quantification of VIP/CR cells, isolated hippocampi double-labeled for CR and GFP in VIP-eGFP mice were analyzed (Fig. 2). As noted above, although the cell bodies and dendrites of many radiatum-targeting VIP-expressing (ISI-2) cells are located in lacunosum-moleculare, a subset have the morphology of ISI-3 bipolar cells, and ∼40% of those express CR (Acsády et al., 1996a), so these counts of VIP+/CR+ cells slightly over-represent the number of ISI-3. Double-labeled GFP(VIP)/CR cell bodies were scattered throughout the pyramidal cell layer and radiatum in control CA1 (Fig. 2*A*) and were preserved in CA1 of epileptic mice (Fig. 2*B*). Quantification confirmed that GFP(VIP)+/CR+ cells were not lost in epileptic mice (1006 ± 88 cells/CA1, *n* = 11 hippocampi from 11 mice) compared with controls (997 ± 92 cells/CA1, *n* = 11 hippocampi from 11 mice, *t* test: *p* = 0.9; Fig. 2*C*). By contrast, GFP(VIP)-positive/CR-negative cell bodies in epileptic mice (343 cells/CA1) were decreased to 54% of controls (632 cells/CA1; *t* test: *p* = 0.0006; Fig. 2*C*). The distribution of GFP(VIP)+/CR+ interneurons along the septo-temporal length of the hippocampus indicated comparable numbers in epileptic mice compared with controls (two-way repeated measures ANOVA: *p* = 0.6 for the septal half, *p* = 0.2 for the temporal half; Fig. 2*D*). However, the distribution of GFP(VIP)+/CR– cells suggested preferential loss at the temporal pole of the hippocampus (two-way repeated measures ANOVA: *p* < 0.001 for the temporal half, *p* = 0.05 for the septal half; Fig. 2*E*).

**Figure 3.**
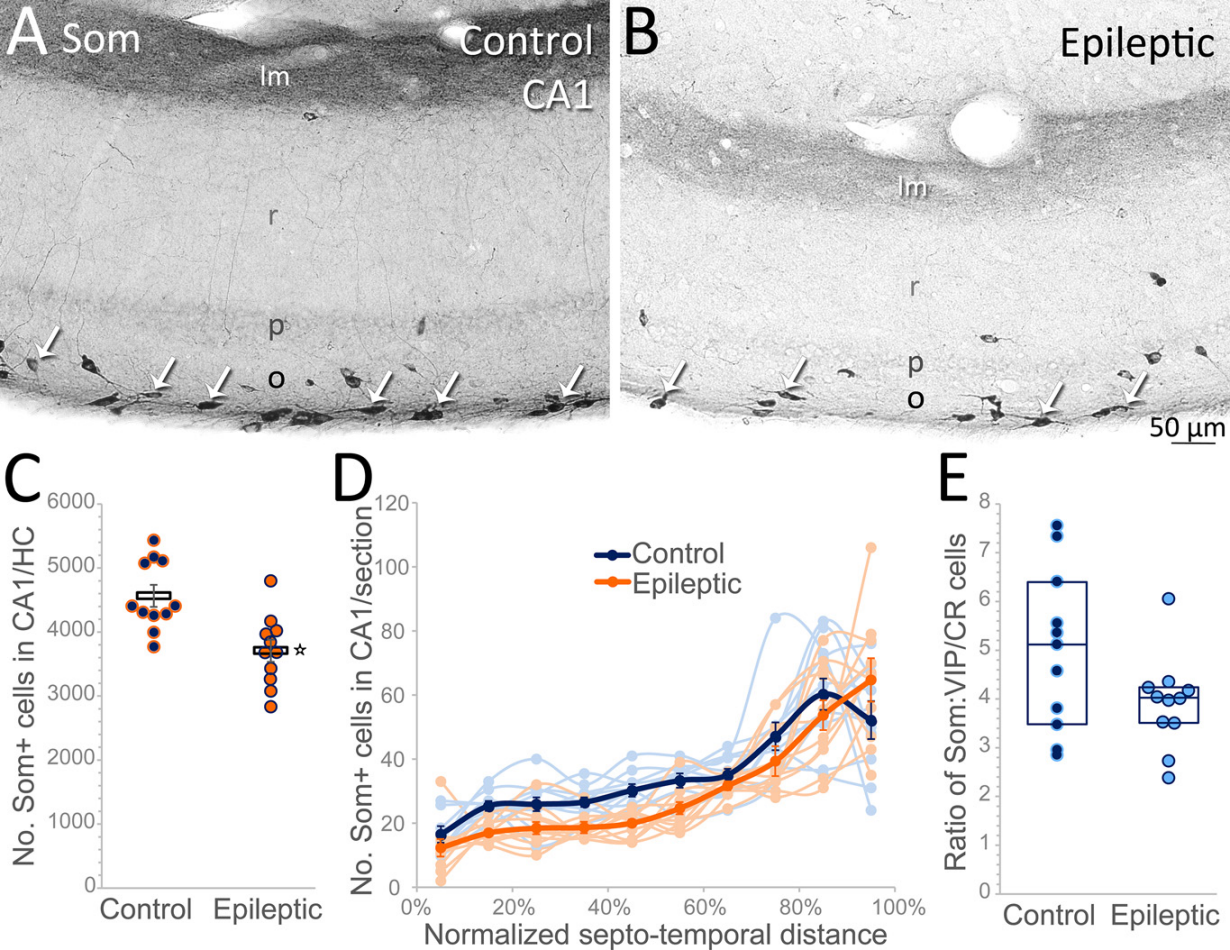
The Som cell targets of VIP bipolar cells in CA1 oriens are reduced in epileptic mice. ***A***, Arrows indicate somatostatin-labeled cells, including OLM cells, in stratum oriens (o) near the alvear border of a control hippocampus. Strongly labeled OLM terminals are evident in strata lacunosum-moleculare (lm). ***B***, Somatostatin-containing interneurons in CA1 oriens are reduced in a section from an epileptic mouse. ***C***, Stereological quantification of the number of somatostatin-labeled interneurons in CA1 oriens confirms a decrease in hippocampi (HC) from epileptic mice (*t test: *p* = 0.001). ***D***, This decrease is biased to septal hippocampal levels. ***E***, Taking each CA1 as a whole, the ratio of Som interneurons to their innervating GFP(VIP)+/CR+ cells is not significantly different between control and epileptic mice (p: pyramidal cell layer, r: stratum radiatum).

Given the sclerosis of epileptic hippocampi, we also quantified the primary target of ISI-3, Som interneurons in CA1 oriens. Somatostatin expression particularly identified cells in oriens near the alvear border, with strong terminal labeling in lacunosum-moleculare consistent with the axonal plexus of OLM cells (Fig. 3*A*). Somatostatin-labeled cells were decreased in epileptic hippocampi compared with controls (Fig. 3*B*), consistent with previous studies (Cossart et al., 2001; Dinocourt et al., 2003; Peng et al., 2013). On average, somatostatin-labeled cells in oriens were reduced by a fifth in epileptic mice (4565 ± 163 cells/control CA1 vs 3709 ± 166 cells/epileptic CA1; *t* test: *p* = 0.001; Fig. 3*C*). Interestingly, the distribution of somatostatin-labeled cells along the septo-temporal axis suggests that loss in epileptic CA1 is greater at septal levels (two-way repeated measures ANOVA: *p* < 0.001 for the septal half, *p* = 0.2 for the temporal half; Fig. 3*D*).

To test whether VIP/CR cells survive disproportionately, which might facilitate hyperinnervation of OLM cells, their hippocampal estimates were compared. The ratio of Som to GFP(VIP)/CR interneurons for each mouse did not show a significant change in epileptic hippocampi (median/25–75% = 4.0/3.5–4.2) relative to controls (median/25–75% = 5.1/3.5–6.4; Mann–Whitney rank-sum test: *p* = 0.1; Fig. 2*E*). However, given that Som cell loss is more pronounced septally, disaggregated analysis indicates that there are fewer Som cells per GFP(VIP)/CR cell in epileptic mice at septal hippocampal levels (septal, temporal: 4.6 ± 0.5, 5.4 ± 0.5 control; 3.2 ± 0.5, 4.5 ± 0.5 epileptic; two-way repeated measures ANOVA: *p* < 0.046 septally, *p* = 0.2 temporally). This indicates an opportunity for VIP/CR cells to increase their inhibition of remaining Som cells in septal hippocampus, but the difference is not great enough to prevail when the hippocampus is considered as a whole. Thus, in epileptic mice VIP/CR bipolar cells persist to inhibit somatostatin-containing cells in a ratio comparable to controls in temporal CA1, and disproportionately outnumber them in septal CA1.

### VIP bipolar cell inhibition of OLM cells

**Figure 4.**
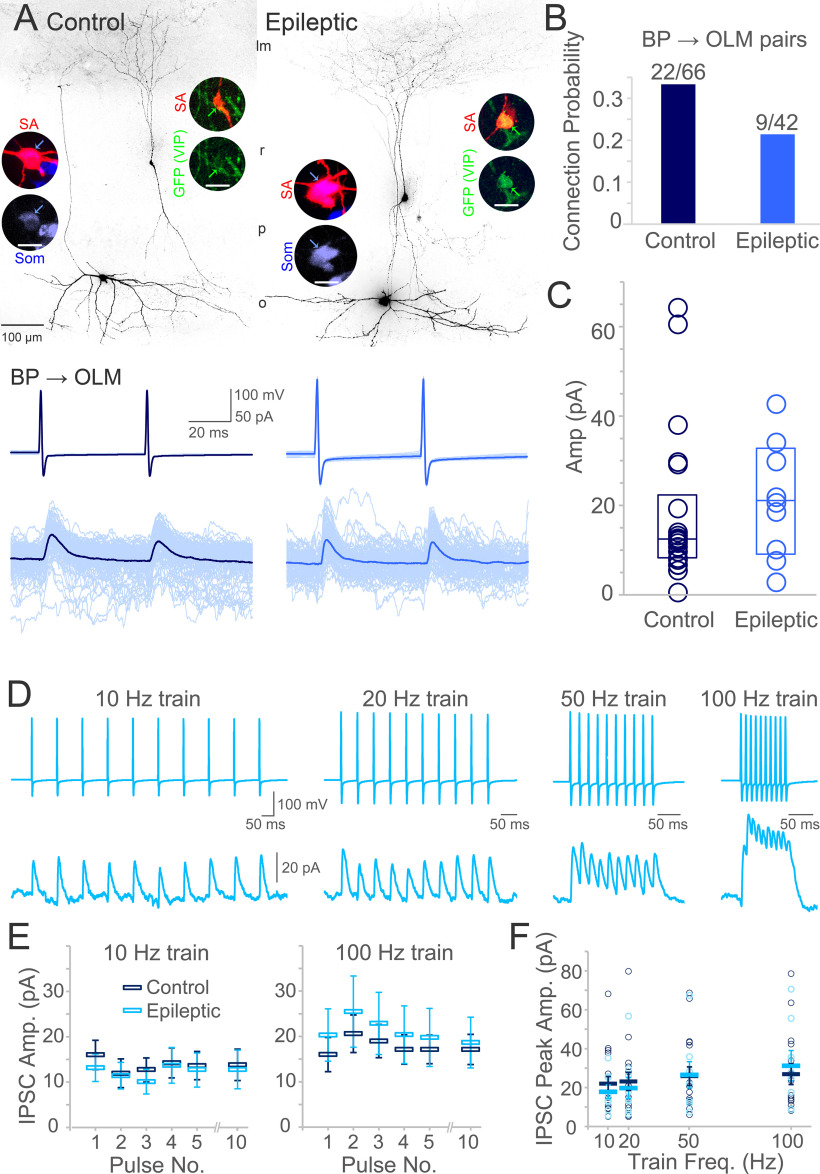
ISI-3 inhibition of OLM cells is not excessive in epileptic CA1. ***A***, Representative examples of recorded VIP bipolar cell (BP) → OLM cell pairs in hippocampal slices. Insets show recorded cells filled with biocytin and labeled with streptavidin (SA; red) and somatostatin (Som; blue) or GFP (VIP, green; scale bar for insets: 20 μm). GFP in particular can be diluted because of the small size of the bipolar cell body. The amplitude of the uIPSC recorded from the epileptic mouse (blue) is not larger than the control pair (navy). The outward inhibitory currents were recorded holding the OLM cell at 0 mV. ***B***, Connection probability between recorded pairs was not increased in pairs from epileptic mice. ***C***, Neither was the average amplitude of the unitary connection significantly increased at the ISI-3 BP → OLM synapse in pairs from epileptic CA1. ***D***, Example trains recorded from a different pair in an epileptic hippocampus. ***E***, On average, IPSC amplitude and summation was not significantly different between epileptic and control pairs. ***F***, Nor was the average peak IPSC amplitude different between epileptic and control pairs (strata oriens: o, pyramidale: p, radiatum: r, lacunosum-moleculare: lm).

The probability or the strength of the connection from ISI-3 may be increased in epilepsy, resulting in hyperinhibition of OLM cells. A recent optogenetic study activated VIP-ChR2 cells and found that the amplitude of light-evoked IPSCs in OLM cells was unchanged in the pilocarpine model, despite the amplitude in oriens basket and bistratified interneurons being reduced (David and Topolnik, 2017). Here, we directly interrogated the VIP bipolar cell to OLM cell synapse with paired recordings in control and epileptic mice. Of 1158 pairs tested, 108 were morphologically confirmed to be ISI-3 and OLM cells (Fig. 4*A*). From control mice, 22 of 66 VIP bipolar to OLM cell pairs were connected (33%), while in epileptic mice 9 of 42 pairs were connected (21%, χ^2^: *p* = 0.3; Fig. 4*B*). This does not support an increase in VIP bipolar cell to OLM cell connectivity in epileptic mice, which could have produced hyperinhibition of OLM cells. Nor were synaptic properties strengthened between the pairs. Measurements of the uIPSC amplitude were not significantly different between the groups, although the statistical power was limited (median/25 75%, control: 12.5/8.3–22.3 pA, epileptic: 21.1/9.1–32.8 pA; Mann–Whitney rank-sum test: *p* = 0.3; Fig. 4*C*). Failure rate was also consistent between groups (33 ± 5% in control and 30 ± 6% in epileptic mice, *t* test: *p* = 0.7). Similarly, the paired pulse ratio between the amplitudes of the second and first pulses at 20 Hz was not significantly different (median/25–75%, control: 1.1/0.9–1.4, epileptic: 0.9/0.8–1.1; Mann–Whitney rank-sum test: *p* = 0.09). IPSC amplitude was further examined during repetitive firing (Fig. 4*D*). On average, IPSC amplitude was not significantly different between groups during trains, although the power was limited (two-way repeated measures ANOVA; Fig. 4*E*), and the peak amplitude was comparable between control and epileptic pairs at various pulse frequencies (two-way repeated measures ANOVA: *p* = 0.2; Fig. 4*F*). Altogether, these data do not indicate hyperinhibition of OLM cells by ISI-3 in epileptic mice because of increased uIPSC amplitude, reduced failure rate, altered release probability, or increased temporal summation.

### Postsynaptic currents in VIP bipolar cells

**Figure 5.**
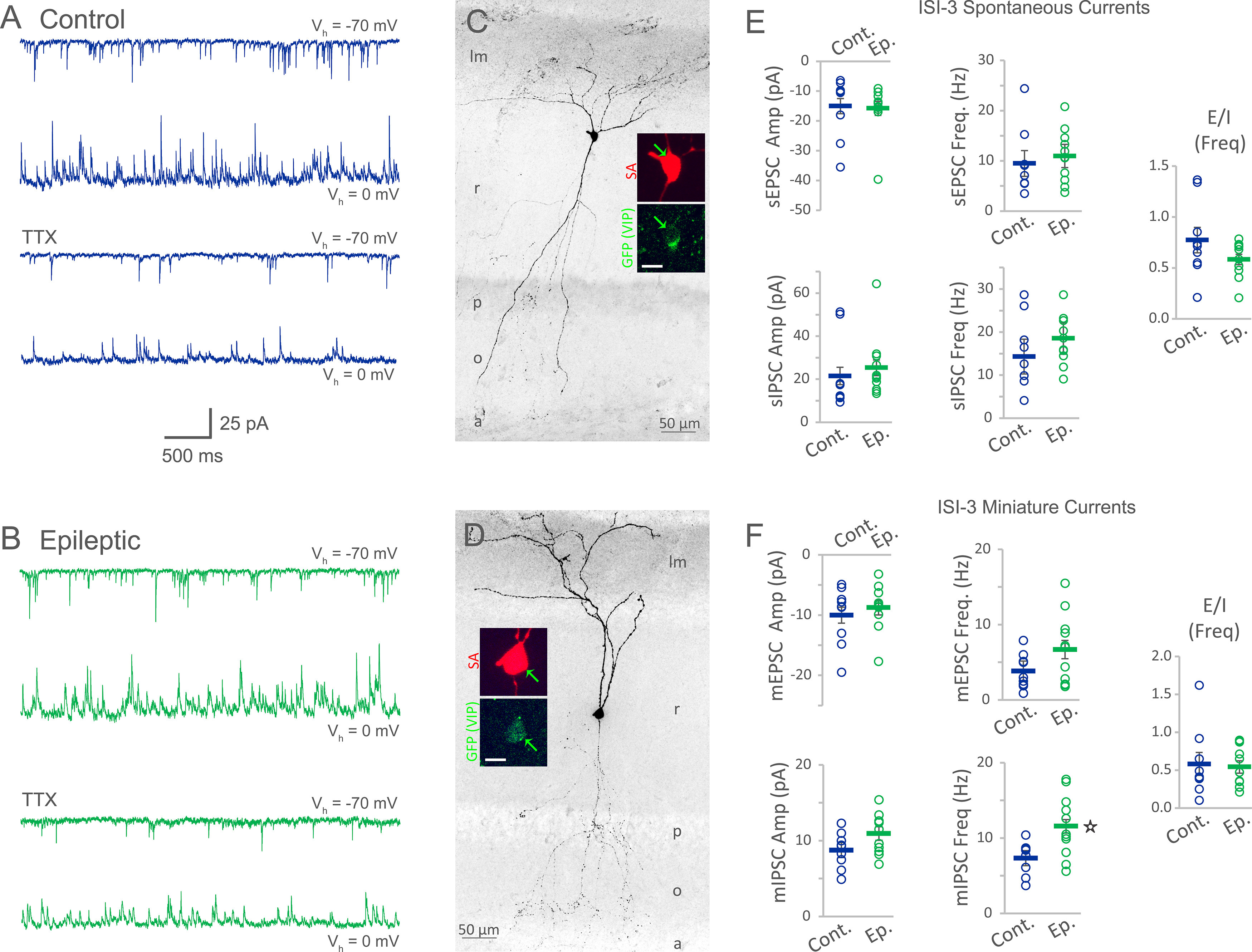
The frequency of postsynaptic currents was largely unchanged in VIP bipolar cells of epileptic mice. ***A***, The first recording shows inward spontaneous EPSCs (sEPSCs) recorded from a GFP(VIP)+ ISI-3 in a slice from a control mouse (navy). The second recording shows outward spontaneous IPSCs (sIPSCs) recorded from the same cell. The third recording shows miniature EPSCs (mEPSCs) following application of 1 μm TTX. The fourth recording shows miniature IPSCs (mIPSCs). ***B***, Postsynaptic currents (green) recorded from a GFP(VIP)+ ISI-3 in a slice from an epileptic mouse under equivalent conditions as the control cell. The currents are similar to control, but mIPSC frequency is increased. ***C***, ***D***, Anatomy for the recorded GFP(VIP)+ bipolar cells from a control mouse (***C***) and an epileptic mouse (***D***) with dendrites extending up through stratum radiatum (r) to strata lacunosum-moleculare (lm) and down (in the case of the control cell) through the pyramidal cell layer (p) to stratum oriens (o), and with light axon labeling in oriens and the alveus (a). Insets show somatic labeling of GFP in VIP-expressing cells (green) colocalized with streptavidin (SA) labeling against biocytin-filled cells (red; scale bars for insets: 10 μm). ***E***, Group data for sEPSCs and sIPSCs indicate that amplitude and frequency were not significantly different between ISI-3 from epileptic mice (Ep.) and controls (Cont.). ***F***, Group data for mEPSC and mIPSC amplitude and frequency indicate that only mIPSC frequency was altered in VIP bipolar cells from epileptic mice (*two-way repeated measures ANOVA: *p* = 0.003). Nevertheless, the ratio for mEPSC frequency to mIPSC frequency (E/I Freq) was unchanged in ISI-3 of epileptic mice.

While ISI-3 inhibition of OLM cells was not significantly different in epileptic mice, it may be that ISI-3 are excessively recruited by the remodeled epileptic circuit, or in advance of a seizure. For insight, we first examined spontaneous currents in VIP bipolar cells with axon in oriens (Fig. 5*A–D*). Neither sEPSC amplitude (−15.0 ± 2.6 pA control, −15.8 ± 2.3 pA epileptic, *p* = 0.8) nor frequency (9.5 ± 2.6 Hz control, 11.0 ± 2.3 Hz epileptic, *p* = 0.7) were significantly different between ISI-3 from each group (nine cells from eight control mice, 11 cells from 10 epileptic mice, two-way repeated measures ANOVA; Fig. 5*E*). Likewise, sIPSC amplitude (21.5 ± 4.0 pA control, 25.3 ± 3.7 pA epileptic, *p* = 0.5) and sIPSC frequency (14.3 ± 4.0 Hz control, 18.5 ± 3.7 Hz epileptic, *p* = 0.4) were similar between VIP bipolar cells in control and epileptic mice (two-way repeated measures ANOVA; Fig. 5*E*). In the presence of TTX to remove action potential-driven events, both mEPSC amplitude (−10.0 ± 1.3 pA control, −8.8 ± 1.2 pA epileptic, *p* = 0.5) and mEPSC frequency (3.8 ± 1.3 Hz control, 6.7 ± 1.2 Hz epileptic, *p* = 0.1) were unchanged in ISI-3 of epileptic mice relative to controls (two-way repeated measures ANOVA; Fig. 5*F*). Neither was mIPSC amplitude altered (8.8 ± 1.0 pA control, 10.9 ± 0.9 pA epileptic, *p* = 0.1). However, mIPSC frequency was increased ∼50% (7.3 ± 1.0 Hz control, 11.6 ± 0.9 Hz epileptic, *p* = 0.003) in VIP bipolar cells from epileptic mice (two-way repeated measures ANOVA; Fig. 5*F*). Nevertheless, comparison of the ratio of mEPSC frequency to mIPSC frequency yielded similar results for cells from control (median/25–75%: 0.4/0.3–0.8) and epileptic mice (0.5/0.3–0.9, Mann–Whitney rank-sum test: *p* = 1.0) suggesting that excitatory and inhibitory inputs remain largely proportional in epileptic VIP bipolar cells (Fig. 5*F*).

**Figure 6.**
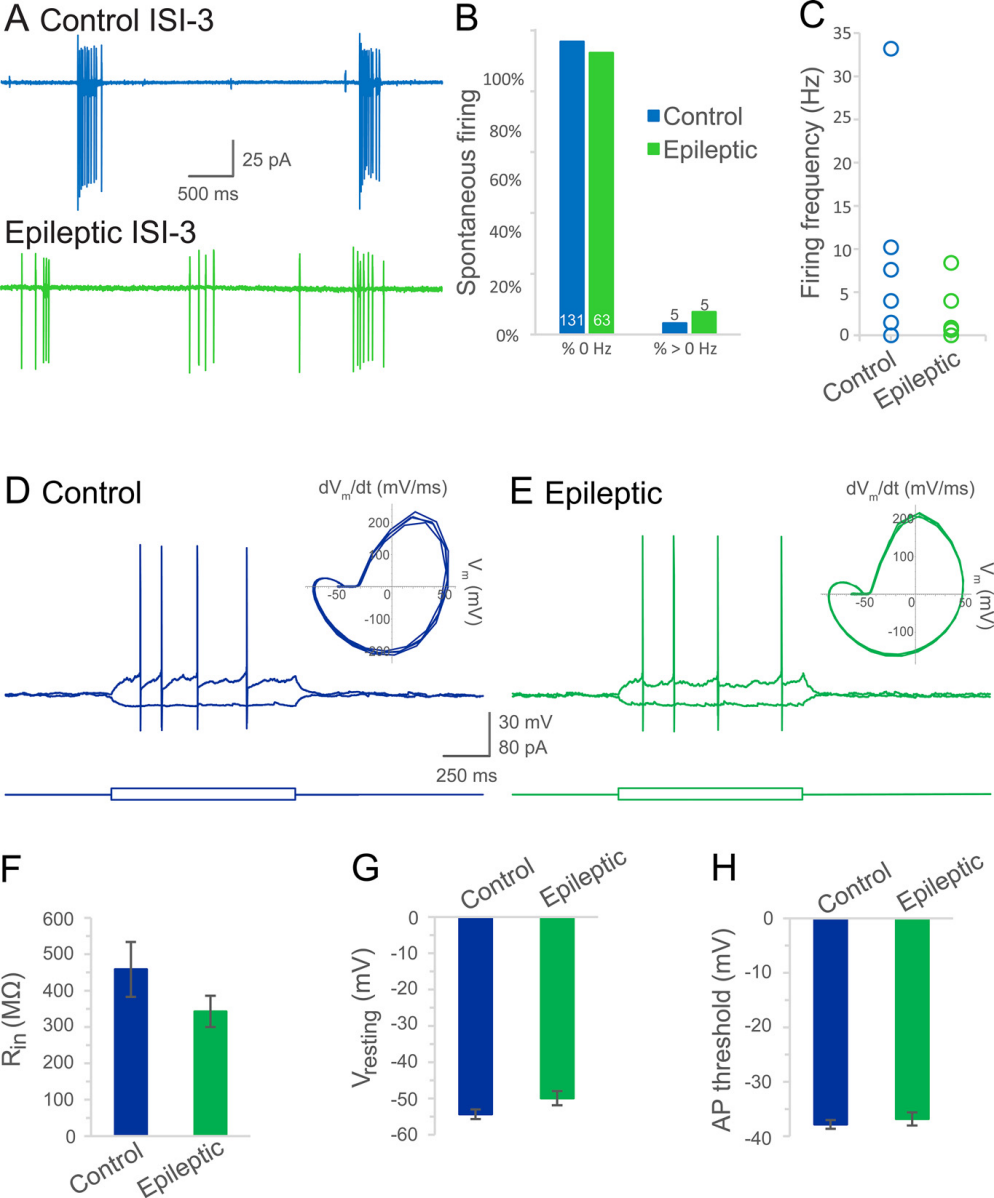
Intrinsic properties of ISI-3. ***A***, Spontaneous action potentials were recorded in cell-attached mode from an ISI-3 of a control mouse (blue) and an epileptic mouse (green). ***B***, Most ISI-3 were not active in either control or epileptic CA1. Values indicate the number of cells in each group. ***C***, Plot of the firing rates recorded in ISI-3 from control and epileptic mice. ***D***, Voltage responses (top) of a different ISI-3 from a control mouse in response to square wave current pulses (bottom, −10 pA and just suprathreshold), and phase plots of the action potentials (inset). ***E*,** Electrophysiological responses (top) from a different ISI-3 of an epileptic mouse in response to current injections (bottom), and phase plots of the action potentials (inset). ***F–H***, Input resistance (R_in_; ***F***), resting membrane potential (V_resting_; ***G***), and action potential threshold (***H***) were not significantly different in the ISI-3 recorded from epileptic mice compared with controls.

Therefore, it is perhaps unsurprising that the action potential firing rate recorded in cell-attached mode from ISI-3s in controls, though low (0.4 ± 0.3 Hz, 134 cells 96% silent), was not significantly different in epileptic mice (0.30 ± 0.2 Hz, 68 cells 93% silent, Mann–Whitney rank-sum test: *p* = 0.4; Fig. 6*A–C*). Intrinsic properties of ISI-3 were also examined to assess changes to excitability in epileptic mice (Fig. 6*D*,*E*). Input resistance was not significantly different between ISI-3 from control or epileptic mice (control: 459 ± 75 MΩ, *n* = 8 cells, 6 mice; epileptic: 342 ± 43 MΩ, *n* = 8 cells, 8 mice; *t* test: *p* = 0.2; Fig. 6*D–F*). Similarly, resting membrane potential in epileptic mice was not significantly different from controls (control: −54 ± 1 mV, *n* = 43 cells, 29 mice; epileptic: −50 ± 2 mV, *n* = 28 cells, 18 mice; *t* test: *p* = 0.06; Fig. 6*G*). Nor was action potential threshold significantly different between VIP bipolar cells from control and epileptic mice (control: −38 ± 1 mV, *n* = 37 cells, 26 mice; epileptic: −37 ± 1 mV, *n* = 25 cells, 18 mice; *t* test: *p* = 0.5; Fig. 6*H*). Altogether, these data do not support a strengthened role for ISI-3 in the epileptic circuit.

### Postsynaptic currents in OLM cells

**Figure 7.**
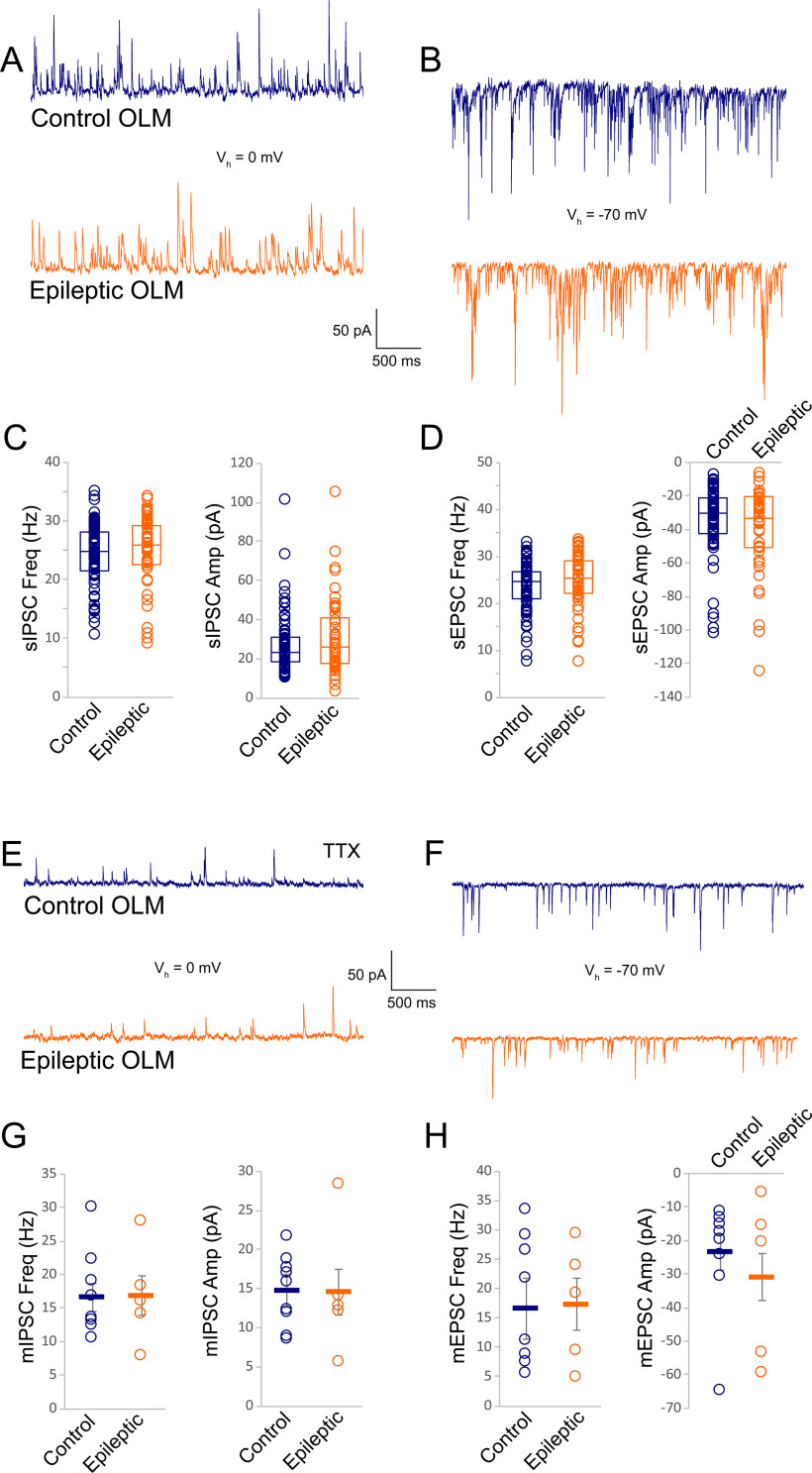
Synaptic inputs to OLM cells were not altered in epileptic mice. ***A***, Slice recordings of sIPSCs in OLM cells from a control (navy) and epileptic (orange) mouse are similar. ***B***, Recordings of sEPSCs from OLM cells are also comparable between the control and epileptic mouse. ***C***, On average, there was no difference in sIPSC frequency or amplitude in OLM cells from control and epileptic mice. ***D***, Neither were sEPSC frequency or amplitude altered in OLM cells of epileptic mice relative to controls. ***E***, ***F***, In the presence of TTX, mIPSCs (***E***) and mEPSCs (***F***) are likewise similar between OLM cells from control and epileptic mice. ***G***, ***H***, Group data indicate that the frequency and amplitude of mIPSCs (***G***) and mEPSCs (***H***) in OLM cells were not significantly different between control and epileptic mice.

**Figure 8.**
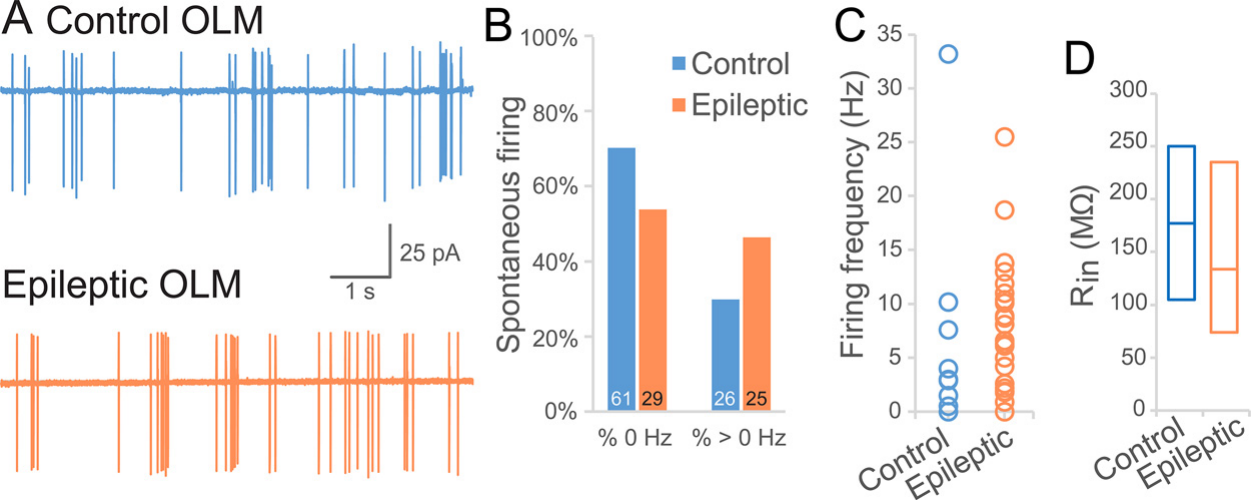
Intrinsic properties of OLM cells. ***A***, Cell-attached recordings of spontaneous action potentials from OLM cells of a control (blue) and an epileptic mouse (orange) are similar. ***B***, Chart of the percentage of OLM cells that were silent or spontaneously firing in control and epileptic mice. ***C***, The action potential frequencies of OLM cells recorded from control and epileptic mice was not significantly different. ***D***, The input resistance of OLM cells recorded from epileptic mice was not significantly different from controls.

In addition to paired recordings between VIP bipolar cells and OLM cells, we examined postsynaptic currents in OLM cells for evidence of hyperinhibition. However, spontaneous inhibitory currents (sIPSCs) in OLM cells were unchanged, either by frequency (median/25–57%, control: 24.8/20.9–28.7 Hz, *n* = 66 cells, 44 mice vs epileptic: 25.9/21.9–29.9 Hz *n* = 48 cells, 30 mice, Mann–Whitney rank-sum test: *p* = 0.3) or by amplitude (median/25–75%, control: 23.0/18.2–31.6 pA, epileptic: 25.9/16.9–41.9 pA, Mann–Whitney rank-sum test: *p* = 0.6; Fig. 7*A*,*C*). Neither were sEPSCs altered, either by frequency (median/25–75%, control: 24.8/20.4–27.3 Hz vs epileptic: 25.3/21.5–29.6 Hz, Mann–Whitney rank-sum test: *p* = 0.2) or amplitude (median/25–75%, control: −30.4/−42.4 to −21.3 pA, epileptic: −33.3/−51.1 to −20.3 pA, Mann–Whitney rank-sum test: *p* = 0.4; Fig. 7*B*,*D*). Likewise, there was no evidence of altered mIPSC frequency in OLM cells (16.6 ± 2.1 Hz, *n* = 9 cells, 8 control mice vs 16.8 Hz ± 2.9 Hz, *n* = 5 cells, 5 epileptic mice) or mIPSC amplitude in OLM cells (control: 14.7 ± 2.1 pA; epileptic: 14.6 ± 2.9 pA, two-way repeated measures ANOVA: *p* = 0.9; Fig. 7*E*,*G*). Nor were mEPSCs altered (amplitude: −23.5 ± 5.2 pA in controls vs −30.9 ± 7.0 pA in epileptic mice, *p* = 0.4; frequency: 16.6 ± 5.2 Hz in controls vs 17.3 ± 7.0 in epileptic mice, two-way repeated measures ANOVA: *p* = 0.9; Fig. 7*F*,*H*). These results do not support an increase in number or strength of individual inhibitory synaptic inputs to OLM cells in epileptic hippocampi. In cell-attached mode, before breaking in, OLM action potential frequency was higher than in ISI-3 (Mann–Whitney: *p* < 0.001) but did not significantly differ between control (2.9 ± 0.6 Hz, *n* = 87 cells, 61 mice, 70% silent) and epileptic mice (4.0 ± 0.8 Hz, *n* = 54 cells, 31 mice, 54% silent, Mann–Whitney rank-sum test: *p* = 0.09; Fig. 8*A–C*). As a gauge of intrinsic excitability, input resistance to OLM cells was not significantly different between OLM cells in control (median/25–75%: 177 MΩ/105–250 MΩ, n = 19 cells, 19 mice) and epileptic mice (134 MΩ/74–235 MΩ, *n* = 17 cells, 13 mice, Mann–Whitney rank-sum test: *p* = 0.3; Fig. 8*D*). Together, these results suggest normal VIP bipolar cell to OLM cell circuit function at baseline.

## Discussion

In order to better understand why OLM cells do not increase their firing rate along with CA1 pyramids in the minutes before a seizure, this study was designed to test the hypothesis that ISI-3 (VIP/CR bipolar cells with axon in oriens) excessively inhibit OLM interneurons in mice with temporal lobe epilepsy. The principal finding is that inhibition of OLM cells by ISI-3 was not strengthened in the pilocarpine model, nor were excitatory inputs to these VIP bipolar cells.

Our finding that ISI-3 to OLM cell connection probability, uIPSC amplitude, failure rate and temporal summation were not significantly different in epilepsy complements the report that the VIP cell to OLM cell optogenetically-induced IPSC amplitude is unchanged in the pilocarpine model (David and Topolnik, 2017). That study found fewer VIP/CR-labeled terminals (but not cell bodies) in CA1 oriens, in a reduction that seemed to be in line with the decrease in their Som interneuron targets. Similarly, we did not find a significant difference in the overall ratio of VIP/CR cells to Som cells in CA1 of epileptic mice. Nor did we observe a change in miniature inhibitory currents recorded in OLM cells, consistent with former studies of interneurons at the oriens/alveus border in the rodent models (Morin et al., 1998, 1999; Dugladze et al., 2007; but see (David and Topolnik, 2017). This suggests that inhibitory inputs to OLM cells do not excessively sprout in epilepsy.

The preservation of VIP/CR bipolar cells contrasts with the loss of approximately half of VIP+/CR– somata across the pyramidal and radiatum layers of CA1. VIP+/CR– cells include cholecystokinin-expressing interneurons (Acsády et al., 1996a; Somogyi et al., 2004; Luo et al., 2020) and the loss reported here is consistent with the degeneration of cholecystokinin basket cell boutons, and their reduced collective functional output, in CA1 of epileptic mice (Wyeth et al., 2010; Kang et al., 2021). It is notable that the decrease in VIP+/CR– cell bodies was particularly apparent in temporal hippocampus, a region commonly identified as the seizure onset zone in the pilocarpine model (Toyoda et al., 2013; Wyeth et al., 2020). The 19% reduction in Som cells found in this study is slightly less than the 24% reduction reported in mouse septal CA1 (Peng et al., 2013), and may reflect our quantifying the whole hippocampus, including the temporal pole which appears to have less severe Som cell loss. Other studies, in rat, estimate a larger Som loss in CA1 oriens (42–46%; Cossart et al., 2001; Dinocourt et al., 2003).

The present finding that miniature excitatory events in ISI-3 were unchanged suggests no aberrant recruitment of these interneurons in temporal lobe epilepsy. Similar to David and Topolnik (2017), there was no significant difference to the input resistance of ISI-3 in epileptic mice, while the resting membrane potential and action potential threshold of ISI-3 were not changed in this cohort. Thus, these data do not suggest increased excitability of VIP bipolar cells in epilepsy, in keeping with the finding that ISI-3 spike frequency was unaltered. Nor was a change in global inhibitory currents in OLM cells evident in this study. The cause of increased mIPSC frequency in VIP bipolar cells here is uncertain, they receive inhibitory inputs from other CR and VIP cells (Acsády et al., 1996a; Freund and Buzsáki, 1996; Gulyás et al., 1996), and septal interneurons (Papp et al., 1999). But connections to VIP cells from Som cells are common and strong in the neocortex (Karnani et al., 2016b) and it is intriguing to consider the possibility that increased inhibitory currents result from reciprocal innervation by OLM cells, which have been shown to sprout in the pilocarpine model (Peng et al., 2013). Increased inhibition to VIP bipolar cells in epilepsy may reflect an effort to compensate for hyperexcitation, e.g., OLM cells may strive both to disinhibit themselves and increasingly inhibit principal cells.

Yet the preictal unresponsiveness of OLM cells spurred this investigation. Although OLM cells are reported to have reduced mEPSC frequency in the intrahippocampal kainate model (but increased sEPSC frequency, Dugladze et al., 2007), here excitatory inputs to OLM cells were unchanged, again in line with previous studies of interneurons at the oriens/alveus border (Morin et al., 1998, 1999; Perez et al., 2006). There is evidence that the input resistance of OLM cells is strongly reduced in temporal lobe epilepsy (Pothmann et al., 2019), lowering their ability to be synaptically recruited; however, input resistance was not significantly different in this cohort of cells (also Morin et al., 1998) and was increased in another study (Dugladze et al., 2007). The recent finding that the facilitating excitatory recruitment of OLM cells by CA1 pyramids is unaltered in the rat pilocarpine model (Pothmann et al., 2019) further suggests that the ISI-3 and pyramidal cell aspects of OLM inputs remain operational in epilepsy.

Several possibilities remain for why OLM cells may fail to increase their firing rate along with CA1 pyramidal cells in the run-up to a seizure. As VIP bipolar cells’ and CA1 pyramidal cells’ dendrites span the same layers of the hippocampus, it might be they are both recruited preictally and that ISI-3 inhibition of OLM cells increases as part of normal circuit function. In support, both Schaffer-collateral and temperoammonic inputs to hippocampal ISI-3 facilitate and effectively recruit VIP bipolar cells (Luo et al., 2020; in contrast with excitatory inputs to neocortical VIP cells which depress; Karnani et al., 2016b). Also, although ISI-3 are less active than other neurons, they are activated by theta frequencies (Luo et al., 2020) which often precede seizure onset in rats (Sedigh-Sarvestani et al., 2014). Furthermore, in neocortex VIP cells have the ability to recruit other VIP cells, partially through nicotinic acetylcholine receptors (Karnani et al., 2016b), and the cooperative firing could provide a way for VIP cells to amplify their activation (Granger et al., 2020). Thus, normal recruitment of ISI-3 to inhibit OLM could be disadvantageous in an epileptic network. Alternatively, there may be a pathologic explanation for the preictal unresponsiveness of OLM cells. For one, there is evidence for degeneration of septohippocampal projections in epilepsy (Wang et al., 2020) and both the cholinergic and GABAergic septal inputs to Som cells and pyramidal cells are suggested to be protective against evoked seizures (Takács et al., 2018; Wang et al., 2020). Additionally, there are a number of metabotropic receptors that may play a vital role. For example, activation of presynaptic GABA_B_ receptors during γ oscillations suppresses transmission between OLM cells and pyramidal cells (Booker et al., 2020), and may similarly suppress OLM cell transmission as global CA1 activity escalates to a seizure. Finally, OLM cells also target bistratified cells and radiatum interneurons to disinhibit Schaffer-collateral inputs to pyramidal cells (Leão et al., 2012), thus the restrained firing of OLM cells may serve to sustain inhibition of Schaffer-collateral inputs. However, unlike principal cells in CA1, the dentate gyrus, and subiculum, CA3 pyramidal cell firing does not increase in the minutes before a seizure (Fujita et al., 2014), and the bulk of OLM axon targets lacunosum-moleculare.

As in other regions of the network, uncertainties remain about the dynamic function of this portion of the epileptic circuit leading up to and during a seizure. As this study rules out chronic hyperinhibition from VIP bipolar cells in epileptic mice, new studies are yet required to determine the reasons underpinning the failure of OLM interneurons to respond at the critical juncture when preictal activity is ramping up.

## Synthesis

Reviewing Editor: Jean Christophe Poncer, INSERM Decisions are customarily a result of the Reviewing Editor and the peer reviewers coming together and discussing their recommendations until a consensus is reached. When revisions are invited, a fact-based synthesis statement explaining their decision and outlining what is needed to prepare a revision will be listed below. The following reviewer(s) agreed to reveal their identity: Katalin Toth.

This study aims to elucidate why OLM interneurons do not increase their discharge along with CA1 pyramidal cells during the preictal period. Understanding the biological basis of this lack of preictal recruitment of OLM interneurons may help to better understand the mechanisms of ictogenesis and provide new avenues for therapeutic intervention.

Specifically, the authors tested the hypothesis that abnormal inhibition of OLM interneurons by ISI-3 interneurons may be responsible for silencing of the former during the buildup of preictal activity. Although this hypothesis is strong and justified, it is not supported by the experimental results of the study, which found no evidence of increased inhibition of OLMs by ISI-3 interneurons.

Both reviewers believe that the study was nonetheless carefully designed and that, even if the hypothesis was found to be false, it provides valuable information in the field. Both reviewers also acknowledge that the experiments were technically challenging and well executed, providing high quality data. They have only minor suggestions to help strengthen the manuscript.

1. The authors provide data on miniature EPSC/IPSCs in OLM and ISI-3 cells, which are informative regarding quantal content and quantal size of synaptic inputs onto these cells. However, spontaneous EPSC/IPSCs may provide additional information regarding interneuron activity in control versus epileptic mice. Have the authors collected such data?

2. Regarding ISI-3 cell excitability, the authors may provide additional information - if available - beyond their firing rate, including input resistance, resting membrane potential, action potential threshold... At least, they should better discuss the possibility that enhanced intrinsic excitability of VIP interneurons in epilepsy may account for the reduced activation of OLM interneurons.

3. The authors sometimes refer to data in the text that are not shown in the figures. These include firing rate of ISI-3 cells, sIPSC/sEPSCs in OLM cells, intrinsic excitability of OLM cells. All data referred to in the Results section should be properly illustrated in the figures.

4. Figure 1 is a bit oversimplified. OLM cells also inhibit PV interneurons that target the pyramidal cell dendrites that receive the Schaffer collaterals (Leao et al. Nat Neurosci 2012). Also, g refers to intrinsic excitability of VIP ISI-3 cells whereas, in the rest of the manuscript, membrane excitability is only evaluated in OLM cells (see point 2).

5. Whereas the Methods section indicates that post hoc verification of interneuron identity was performed using immunofluorescent staining of GFP and SOM, data presented in Fig. 4 and 5 only display biocytin labeling. Why?

6. Page 3, please better define Dlx-1.

## Author Response

Response to Reviewers,

Thank you for reviewing our manuscript. We appreciate the helpful comments. The revisions we made improved the manuscript and we address the particular points below.

1. The authors provide data on miniature EPSC/IPSCs in OLM and ISI-3 cells, which are informative regarding quantal content and quantal size of synaptic inputs onto these cells. However, spontaneous EPSC/IPSCs may provide additional information regarding interneuron activity in control versus epileptic mice. Have the authors collected such data?

We added the data collected on spontaneous currents in ISI-3 to the manuscript (page 12, paragraph 2) and Figure 5, and to the spontaneous currents in OLM cells to Figure 7.

2. Regarding ISI-3 cell excitability, the authors may provide additional information - if available -

beyond their firing rate, including input resistance, resting membrane potential, action potential

threshold... At least, they should better discuss the possibility that enhanced intrinsic excitability of

VIP interneurons in epilepsy may account for the reduced activation of OLM interneurons.

We agree on the importance of enhanced intrinsic excitability as a factor, and found no change here. We pulled the ISI-3 input resistance and resting membrane potential out of the text and added action potential threshold, creating Figure 6. It is discussed on page 16, paragraph 2.

3. The authors sometimes refer to data in the text that are not shown in the figures. These include

firing rate of ISI-3 cells, sIPSC/sEPSCs in OLM cells, intrinsic excitability of OLM cells. All data

referred to in the Results section should be properly illustrated in the figures.

We created Figure 6 to illustrate the firing rate of ISI-3 cells and their intrinsic properties, we added the spontaneous currents in OLM cells to the Figure 7, and we created Figure 8 to illustrate the firing rate and input resistance of OLM cells.

4. Figure 1 is a bit oversimplified. OLM cells also inhibit PV interneurons that target the pyramidal

cell dendrites that receive the Schaffer collaterals (Leao et al. Nat Neurosci 2012). Also, g refers to

intrinsic excitability of VIP ISI-3 cells whereas, in the rest of the manuscript, membrane excitability is only evaluated in OLM cells (see point 2).

The reviewers are right to point out that OLM cells inhibit PV/bistratified cells and radiatum interneurons, and this was not included in the admittedly simplified diagram in Figure 1. Indeed VIP cells also inhibit PV interneurons to a degree. However, this study really focused on why OLM cells do not ramp up their firing preictally, testing the hypothesis that it is due to enhanced inhibition of OLM cells by VIP interneurons, and we did not address any changes to PV interneurons. If the reviewers would permit, we would like to leave the diagram in this simplified state to help readers understand the focus of the study. We added the reviewers’ point to the discussion (page 18, paragraph 1). The intrinsic excitability of VIP ISI-3 got lost in the text, so we pulled it into Figure 6.

5. Whereas the Methods section indicates that post hoc verification of interneuron identity was

performed using immunofluorescent staining of GFP and SOM, data presented in Fig. 4 and 5 only

display biocytin labeling. Why?

We did stain all the recorded cells for GFP and Som, but in the end it became clear that the critical feature for identification of both OLM and ISI-3 cells was the location of the axon in slm or oriens respectively (we tried to highlight that with the adjectives ‘distinctive’ and ‘distinguishing’ in the methods on page 8, paragraph 2, and ‘morphologically confirmed’ in the results on page 11, paragraph 2). Showing all the labeling in the figures made the fine axons even harder to see, but we have added the somatic labeling in insets to Figures 4 & 5. Some of the protein was diluted during recording, but we hope that our remedy addresses the reviewers’ comment.

6. Page 3, please better define Dlx-1.

We added text to define and explain Dlx1 at the top of page 3.
